# Biophysical and biochemical aspects of immune cell–tumor microenvironment interactions

**DOI:** 10.1063/5.0195244

**Published:** 2024-04-02

**Authors:** Shoham Benmelech, Thien Le, Maggie McKay, Jungmin Nam, Krupakar Subramaniam, Daniela Tellez, Grace Vlasak, Michael Mak

**Affiliations:** 1 Department of Biomedical Engineering, Yale University, New Haven, Connecticut 06511, USA; 2 Department of Molecular, Cellular, and Developmental Biology, Yale University, New Haven, Connecticut 06511, USA

## Abstract

The tumor microenvironment (TME), composed of and influenced by a heterogeneous set of cancer cells and an extracellular matrix, plays a crucial role in cancer progression. The biophysical aspects of the TME (namely, its architecture and mechanics) regulate interactions and spatial distributions of cancer cells and immune cells. In this review, we discuss the factors of the TME—notably, the extracellular matrix, as well as tumor and stromal cells—that contribute to a pro-tumor, immunosuppressive response. We then discuss the ways in which cells of the innate and adaptive immune systems respond to tumors from both biochemical and biophysical perspectives, with increased focus on CD8+ and CD4+ T cells. Building upon this information, we turn to immune-based antitumor interventions—specifically, recent biophysical breakthroughs aimed at improving CAR-T cell therapy.

## I. THE TUMOR MICROENVIRONMENT

The tumor microenvironment (TME) encompasses cancer cells, genetically non-cancerous cells, and the extracellular matrix (ECM). The TME plays a pivotal role in cancer progression, and its malignant phenotype is influenced by its dimensionality, topography, and porosity.^1^ Here, we discuss three relevant dimensions of the TME: physical, mechanical, and cellular.

### A. Physical aspects of the TME

The physical components of the TME can be further understood as a set of complex interactions between spatial, temporal, and topographical dimensions. Spatial dimensionality refers to the physical arrangement of cells and components within the TME.^2^ Tumor cells interact with stromal cells, immune cells, blood vessels, and the ECM in a spatially organized manner. The three-dimensional (3D) structure of the TME is also crucial for understanding cell–cell interactions, nutrient and oxygen gradients, and therapeutic delivery.^1,2^

Given the dynamic processes occurring within the TME (i.e., angiogenesis, immune cell infiltration, and ECM remodeling), temporal dimensionality is another essential facet. The ECM is a dynamic tissue support network that has implications for cellular proliferation, differentiation, and tissue homeostasis.^1,3^ It is composed of two main classes of macromolecules: proteoglycans (hydrated gels that fill most of the extracellular interstitial space) and fibrous proteins (primarily, collagen, fibronectin, elastin, and laminin).^3^ Collagen can form fibrils, beaded filaments, and networks by intermolecular cross-linking, while fibronectin connects structural proteins (such as collagen) to form an integrated matrix.^4^ Fibronectin has additional effects on cell adhesion, migration, proliferation, and vascularization.^4,5^ Elastin provides elasticity to the network, thereby protecting it against tissue deformation and rupture, while laminin, along with collagen, constitutes the basement membrane and is involved with vessel maturation.^6–8^

**FIG. 1.**
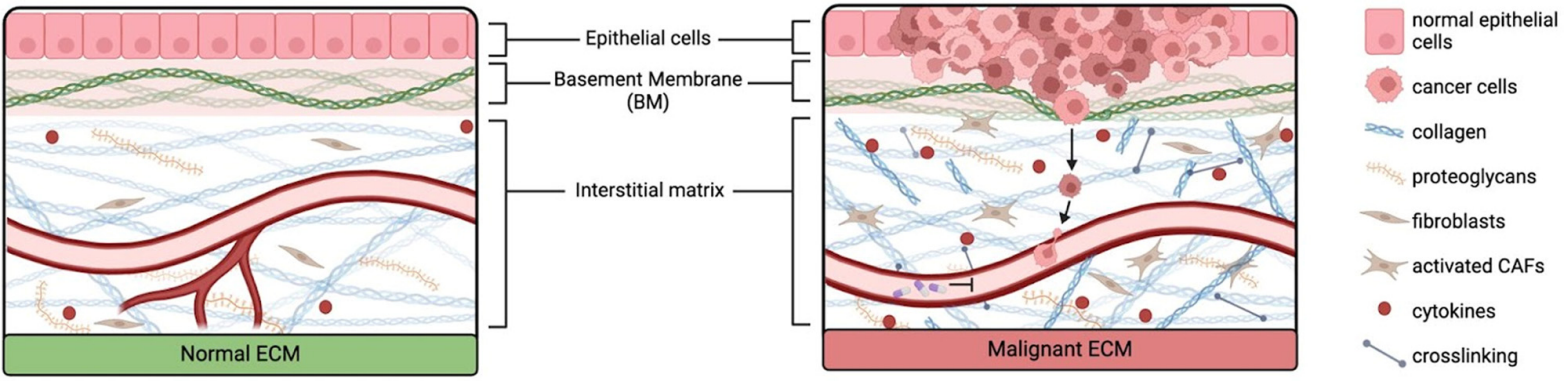
Normal and malignant ECM and their differences in biophysical, mechanical, and cellular components. Malignant ECM contains increased collagen fiber deposition and collagen cross-linking, cytokine release, activated fibroblasts (here, cancer associated fibroblasts or CAFs), and cancer cell growth. These factors lead to increased stiffness and altered porosity and architectures. Malignant ECM has decreased vasculature, limiting drug delivery. Cancerous epithelial cells can more easily traverse the basement membrane and enter the vasculature.

The topography of the TME refers to the physical features and characteristics of the surroundings in which a tumor exists (Fig. 1). It encompasses spatial arrangements, structural elements, and the overall landscape that influences interactions between cancer cells and their surrounding microenvironment.^1^ The porosity of the TME refers to the extent to which the tissue allows the movement of fluids, molecules, and cells within its structure and plays a significant role in various biological processes associated with tumor growth, invasion, and response to therapy. Collagen density strongly influences matrix porosity and stiffness (described in Sec. I B), which can modify cell behaviors, such as migration, proliferation, differentiation, and apoptosis.^1^

### B. Mechanical aspects of the TME

The mechanical properties of the TME are important in the determination of cell behavior. Following malignant transformation, the TME becomes mechanically complex due to ECM mechanical reprogramming.

The main mechanical component of the TME is the stiffness of the ECM. ECM stiffness, also referred to as matrix stress, is the result of changes in the cross-linking of ECM proteins and increased deposition of compounds such as collagen into the ECM.^9^ Biological effects of matrix stiffness on cancer cells include proliferation, metastasis and mobility, therapeutic resistance, and abnormal angiogenesis.^9^ Matrix stiffening contributes to cancer cell proliferation through multiple signaling pathways, including the canonically relevant Hippo pathway, which involves Mammalian Ste20-like kinases (MST1/2), large tumor suppressor kinases (LATS1/2), and effectors Yes-associated protein (YAP) and transcriptional coactivator with the PDZ-binding motif (TAZ).^10^ In the context of cancer, dysregulation of the Hippo pathway can lead to overactivation of YAP and TAZ, resulting in uncontrolled cell proliferation.^10^

Cancer cells also tend to exhibit greater mobility on a stiffened ECM. The stiffness of the ECM can directly activate several signaling transduction pathways involved in cell migration—for example, Rho/Rho-associated coiled-coil containing protein kinase signaling is activated when cytoskeletal tension is increased in a stiffened matrix.^11^ At the same time, collagen-induced integrin clustering induces the recruitment of focal adhesion signaling molecules, such as FAK, Src, paxillin, Rac, Rho, and Ras, eventually promoting the contraction and progression of cancer cells.^11^ In 3D collagen-alginate hydrogels, increased ECM stiffness has been demonstrated to disrupt cell volume homeostasis in migrating cancer cells. The stiffened tumor matrix also decreases drug sensitivity by forming a physical barrier against drug infiltration into the tumor tissue. A stiff matrix compresses micro blood vessels, making it difficult for drugs to access core tumor tissues via the vasculature.^11^

Vascularization can also be an indicator of tumor development and progression. During angiogenesis, the matrix of the basement membrane is synthesized while vascular loops—which link the parental vessels and the neovessels—are formed; however, increasing the density of both collagen and fibrin matrices has been shown to decrease vascularization.^11^

### C. Cellular aspects of the TME

In addition to the ECM, another key component of the TME is cells, which contribute to cancer progression, build the ECM, and dictate the biochemical environment.^12^ Here, we review the role of the stroma in cancer progression as well as the heterogeneity of tumor cells.

#### 1. The stroma

The stroma consists of cells within the tumor that are not cancer cells themselves. These include fibroblasts, endothelial cells, pericytes, and immune cells, each of which can take on an activated, cancer-associated phenotype that aids in cancer progression.^13^

A key component of the stroma is cancer associated fibroblasts (CAFs): activated fibroblasts that contribute to tumorigenesis in a multitude of ways. They can originate not just from fibroblasts, but many different cell types, such as endothelial cells.^14,15^ CAFs affect both the biophysical TME through fibrin deposition and the biochemical TME by secreting factors such as cytokines and growth factors that contribute to cancer plasticity and immune suppression^16–18^ (Fig. 1**)**. A recent work has also tried to connect the presence of CAFs with patient outcomes. In particular, Gu *et al.* found that a high fibroblast-related score (FRS) was associated with decreased patient survival, whereas patients with a low FRS were more susceptible to immunotherapies, supporting the importance of CAFs in immune evasion.^19^

Crosstalk between the tumor and stroma—that is, signals transferred between cancer cells and CAFs—has also been a significant area of recent research. When looking at the ability of pancreatic cancer to become resistant to gemcitabine drug treatment, Qi *et al.* found that only when cancer cells were cocultured with CAFs did the exosomes released from CAFs initiate drug resistance.^20^ Another group showed an important crosstalk axis where CAFs secrete IL-6, and breast cancer cells develop radioresistance through CAF mediated activation of the STAT3 pathway; blocking this IL-6-STAT3 axis decreased breast cancer growth and increased susceptibility to treatment.^21^ This two-way conversation between the tumor and stroma has contributed to the adaptation and cohesiveness of cancer.

#### 2. Tumor heterogeneity

A cancerous tumor is not a uniform population of cancer cells but rather a heterogeneous and intricate community. This heterogeneity occurs on many levels, including between patients diagnosed with the same cancer, intra-tumor, and with cancer stem cell differentiation potential.^22,23^ A hallmark of cancer is its genetic instability, and the unique combination of genetic mutations and biomarkers compounded by tumor heterogeneity increases the difficulty of treatment.^24^

Understanding this diversity is essential for improving treatment plans. Gay *et al.* used transcriptional analysis to characterize four subtypes of small cell lung cancer that were previously considered to be analogous between patients.^25^ Their research showed that certain subtypes are more susceptible to immunotherapy and chemotherapy than others and further found that subtype switching could occur, resulting in a tumor evolving throughout the course of treatment.^25^ Another study characterized cells within different types of breast cancers and found unique biomarkers for each that were used to predict drug resistance and outcomes.^26^ Characterizing the cancer subtype is, thus, an essential step in cancer research to best identify treatment options.

Single-cell RNA sequencing (scRNA-seq) has emerged as a compelling technique to identify tumor heterogeneity. Recently, this technique has been used to explore differences within ovarian, prostate, and brain tumors.^27–29^ As scRNA-seq research continues to evolve, clinicians can better identify specific cancer subtypes and adjust treatment appropriately.

### D. Implications of tumor-associated remodeling

The ECM is continually reshaped by cell–matrix and cell–cell activity during tumor progression through synthesis, degradation, reassembly, and chemical modification; moreover, the capacity of tumor cells to recognize particular motifs in ECM molecules and register a stiffened matrix enhances tumor cell invasion, migration, and differentiation.^9,30^ Crosstalk between cells is often mediated through secreted factors, which contribute to the biochemical environment of the TME. These factors can be broadly categorized into two functions: pro-cancerous and immunosuppressive. Pro-cancerous factors induce disease progression, such as angiogenesis, differentiation, and epithelial–mesenchymal transition (EMT) and invasion.^31–33^ These factors can come from both cancer cells and stroma cells, caused by a variety of stimuli. Factors that allow cancer to evade destruction or—more relevant for this review—evade the immune system are considered immunosuppressive and are from multiple cell sources, including immune cells themselves.^33,34^ The main methods for biochemical immunosuppression are through factors that decrease immune cell recruitment or factors that inhibit antitumor actions.^33,35^ For example, recent work found that cancer cells were able to induce transcriptional changes in T cells in coculture, including an overabundance of *KLK3*, which may be related to micrometastasis.^28,36^

ECM remodeling can occur via changes in macromolecule components, protein activity, stiffness, and increased cross-linking of proteins.^37^ In most tumors, remodeling occurs through increased collagen synthesis and deposition accompanied by remodeling enzymes, such as matrix metalloproteinases (MMPs)—which can degrade collagen and connective tissue—and lysyl oxidase (LOX)—which catalyzes cross-linking of collagen to control matrix stiffness and cell–matrix interactions.^38^

Alterations in expression of ECM components—namely, collagen synthesis and deposition—results in heightened matrix density and serves as a physical barrier for tumor cells against immune destruction: recent work has found that in triple negative breast cancer, high Th1 (helper T cell) infiltration has been related to low collagen I content, whereas high Treg infiltration has been observed in collagen-rich lesions.^39^ Additionally, the hypoxia-related collagen V gene (COL5A1) was overexpressed in a variety of tumor types, including lung, breast, colorectal and gastric cancers, melanoma, liver hepatocellular carcinoma, and prostate adenocarcinoma, suggesting that COL5A1 expression increases during tumor progression.^40^

The TME, thus, represents a dynamic system, with complex physical, mechanical, and cellular components that work in conjunction to improve tumorigenesis (Fig. 1). Additionally important to studies of tumor cells is the interactions between the TME and immune cells. For that reason, we will next focus on recent studies that have elucidated the biochemical and biophysical mechanisms underlying crosstalk between tumor cells and host immune response.

## II. IMMUNE CELL RECRUITMENT TO THE TME

In order to infiltrate the TME, immune cells must interact with biophysical and biochemical components of the TME. Immune cell infiltration is guided by physical limitations of the ECM, cell adhesion sites, and tumor cell blockades, as well as biochemical cues of the ECM and chemokine signaling. The resulting mechanobiology of immune cells dictates the degree to which cells can eliminate or enhance cancer growth. Here, we summarize key components of the innate and adaptive immune response that interact with tumor cells and the TME as well as the biochemical and biophysical aspects underlying these interactions.

### A. Innate immune response

#### 1. Neutrophils

**FIG. 2.**
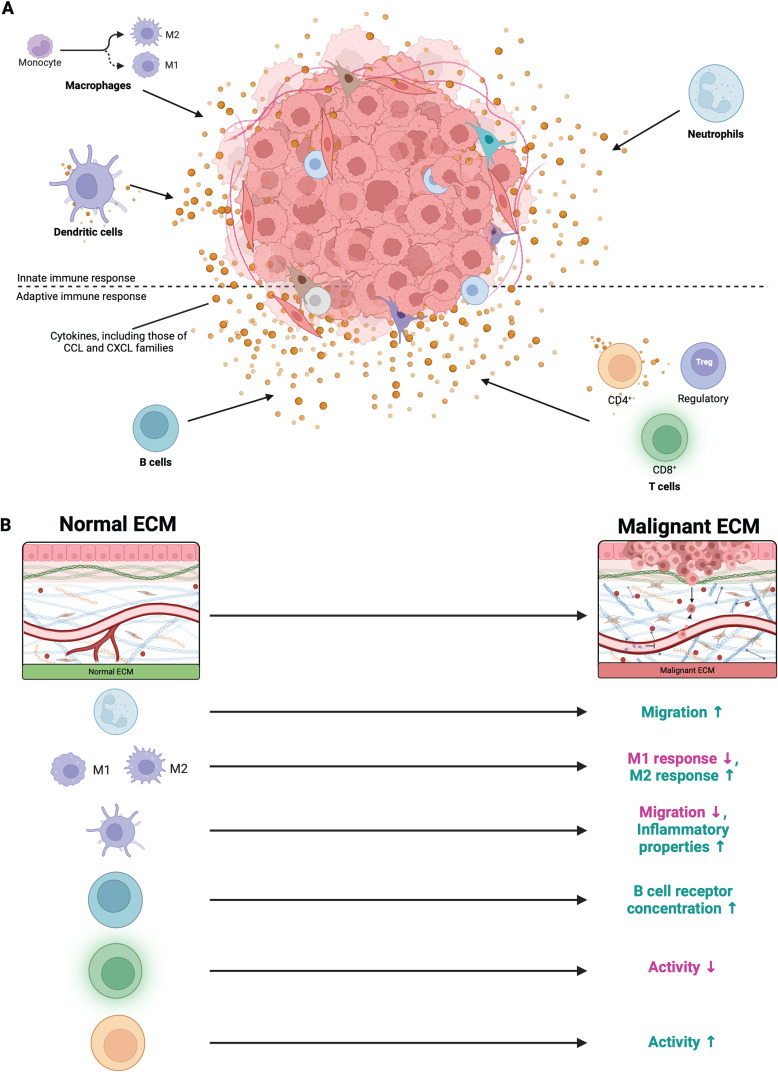
Biochemical and biophysical barriers to recruitment of immune cells to TME. (a) The secretion of cytokines by the tumor promotes recruitment of many cells of both the innate and adaptive immune responses. (b) The biophysical changes associated with an increased tumorigenic ECM affect immune cells in different ways.

Essential to the recruitment of immune cells to a tumor site are chemokines. For neutrophils, in particular, tumor cell expression of CXCL1, CXCL2, and CXCL5 increased neutrophil recruitment to the tumor and blocking CXCR2 signaling has been shown to decrease neutrophil-tumor infiltration^41,42^ [Fig. 2(a)]. Neutrophils are unique, however, in that their recruitment to tumor cells may be associated with either pro-tumor or anti-tumor outcomes.^43,44^ For example, the effect of tumor associated neutrophils (TANs) has been directly tied to the existing state of the tumors for which the TANs are targeted. In early murine tumor stages, TANs were more cytotoxic, producing more TNF-α, whereas TANs developed a pro-tumorigenic phenotype in well-established tumors.^45^ Further complicating the exact role of neutrophils within the TME, poor breast cancer and lung adenocarcinoma prognosis have been associated with high TAN density, while other cancers do not exhibit such an association.^46^ Despite the dual role of neutrophils, the chemical basis for their activity is rooted in chemokine activation and subsequent neutrophil chemotaxis.

There exist, however, biophysical factors that impact their activity; neutrophils are sensitive to changes in substrate stiffness and shear stress. Changes to these environmental parameters can induce activation, depolarization, phagocytosis, and the formation of NETs (neutrophil extracellular traps). At rest, neutrophils traverse the bloodstream and have a spherical form. When activated, they upregulate chemokines and activation markers (such as CD11b, CD66b, and CD64) and express pseudopod projections as they hunt for pathogens. Neutrophils can be activated by the shear force experienced during circulation, as well as deformation that occurs during the extravasation of neutrophils from the bloodstream to the tissue.^47–49^ Once neutrophils reach the tissue, they are able to migrate via adhesion molecules ICAM, V-CAM, and microvilli. While increased matrix stiffness increases neutrophil migration, the mode of migration is not sensitive to increasing substrate stiffness.^50^ The efficacy and directionality, however, are improved with stiffer substrates^51^ [Fig. 2(b)].

#### 2. Macrophages

Similar to neutrophils, tumor associated macrophage (TAM) infiltration is caused by expression of chemokines–specifically, CCL2^52^ [Fig. 2(a)]. Of important note, two major macrophage phenotypes exist: M1, which is pro-inflammatory, and M2, which is crucial for promoting wound healing, tissue repair, and cell proliferation.^53^ Given the differing phenotypes within the body, macrophages are, thus, similar to neutrophils in that their effect on tumors is varied and difficult to predict.

Macrophages are also affected by changes to the mechanical environment. Increasing stiffness coupled with niche organ signals can condition the macrophage to vary subtypes; macrophages primed on a stiffer environment similar to bone acquire a tissue-residency phenotype.^54^ Softer substrates yield organ specific macrophages. Physical confinement of the macrophage, as seen in substrates with small porosity, can downregulate the M1 response in macrophages [Fig. 2(b)]. Increased interstitial flow has been observed to increase the M2 phenotype.^55^

#### 3. Dendritic cells

Dendritic cell (DC) recruitment is mediated by various chemokines; for example, TMEs lacking CCL4 recruited fewer CD103+ DCs.^56^ Moreover, dendritic cells themselves release CCL2 and other chemokines that recruit additional dendritic cells, natural killer cells, and T cells, serving as a bridge to the adaptive immune response^57–59^ [Fig. 2(a)]. Chemically, dendritic cells serve an important role in communicating tumorigenesis to other components of the innate and adaptive immune response.

However, biophysical changes can affect dendritic cell behavior. Environmental stiffness can promote inflammatory DC phenotype, while increased cellular tension impacts DC metabolism.^60^ While promoting inflammatory DCs, increased stiffness and compression also leads to consistently slower DC migration/motility^61^ [Fig. 2(b)]. Cyclic mechanical strain has been shown to adversely affect DC viability without affecting cytokine profiles.^62^

### B. Adaptive immune response

As previously demonstrated, the TME is a heterogeneous composition of not only tumor cells and ECM proteins but also components of the innate immune system. Also important to tumorigenesis is the role of the adaptive immune response. Specifically, prior work has demonstrated a reduction in tumor suppressor genes in immunologically healthy mice relative to immunocompromised mice.^63^ Moreover, there is significant interplay between the innate and adaptive immune responses.

At the same time, there are conflicting results regarding the effect of tumor-derived ISGs on adaptive immune cell recruitment. For example, research on nasopharyngeal carcinoma cells demonstrated that ISG15 expressed in cancer cells limited the adaptive immune response and promoted tumor progression, while other studies have found that STING activation led to IFN production and subsequent conversion of monocytes from immunosuppressive to immunostimulatory as well as upregulation of chemokines, such as CCL5 and CXCL10.^54,64,65^

The interconnectedness between the innate and adaptive immune responses, as well as the role of the adaptive immune response itself in regulating tumorigenesis, warrants a more comprehensive look at both the recruitment of adaptive immune cells to the TME and the phenotype of T cells subsequently localized to this area.

#### 1. B cells

One crucial component of the adaptive immune response is B cells, which have been implicated in both tumor-suppressive and tumor-promotive processes.^18^ Moreover, documented evidence of B cells infiltrating cancers ranging from the lungs to the pancreas exists, highlighting the relevance of B cells to the field of cancer immunotherapy.^66^

There are two principal mechanisms through which B cells are recruited to tumors. First, B cells are attracted to a tumor location due to the release of cytokines and chemokines, such as those of the CC family.^18^ A second, more recently discovered method by which B cells accumulate at a tumor site is through TANs. Specifically, TANs recruit B cells to a tumor site through TNFα and are responsible for subsequent differentiation into CD138+ IgG-producing plasma cells.^66^

A relevant factor in informing B cell phenotype is interactions with antigen presenting cells and T cells. These cells have varying topography and stiffness that are sensed by B cells and offer distinct differences in subsequent B cell behavior. Additionally, MHCII, CD80/86, and CD40 direct B cell/T cell interactions and facilitate different B cell pathways.^67^

B cell cytoskeletal changes occur during antigen-B-cell receptor (BCR) binding and contract the B cell. This increases cell spreading and endocytosis. Actin filaments similarly respond and control kinase/phosphatase accessibility on the surface of the cell. Different topographical patterns can be sensed by B cells via PKCβ and focal adhesion kinases (FAKs).^68^ Higher stiffnesses result in FAK activation, increased spreading, and adhesion to antigen-presenting cells (APCs). Stiffer substrates have been shown *in vitro* to promote more BCR accumulation^67^ [Fig. 2(b)].

#### 2. CD8+ T cells

A critical component of the adaptive anti-tumor response is cytotoxic CD8+ T cells, with importance further underscored by research demonstrating that blocking PD-1/PD-L1 signaling in tumor cells enhances CD8+ T cell infiltration and combats tumor progression.^69,70^ As shown above in discussing components of the innate immune response in the context of tumorigenesis, chemokines play a dual role in the body; they are necessary for the recruitment of immune cells to particular areas of the body, but they may also induce pro-tumor activity. For example, CCL21 and CCL4 have been shown to activate T cells while simultaneously recruiting Tregs, which have immunosuppressive properties and are discussed in more detail below.^46,71–73^ Further evidence of the importance of chemokines to recruitment of CD8+ T cells stems from CRISPR-mediated knockout of STING in cancer cells, which limited T cell infiltration *in vivo*.

Work on non-small cell lung cancer has shown that inhibiting mitogen-activated protein kinase kinases (MEKs) triggers CXCL10 secretion, resulting in recruitment of CD8+ T cells presenting with CXCR3 [Fig. 2(a)]; conversely, activation of components of the hedgehog pathway in tumor cells has been shown to promote TAM M2 polarization, thus downregulating expression of CXCL9 and CXCL10 and limiting CD8+ T cell recruitment.^74–77^ Moreover, researchers found that treatment with chemotherapeutic compound docetaxel is associated with higher expression of CXCL11, greater infiltration into the TME of CD8+ T cells, and improved lung cancer prognosis.^78^ These observations not only demonstrate the crosstalk between innate and adaptive immune cells but also the importance of chemokines of the CXCL family to CD8+ T cell recruitment.

Once in the TME, CD8+ T cells differentiate into their classic cytotoxic phenotype, resulting in additional secretion of cytokines and memory CD8+ T cells; however, the TME contributes to suppression of these traditionally cytotoxic CD8+ T cells.^79,80^ This phenotypic change is part of larger observations of T cells converting to an exhausted state within the TME, limiting immunological function and enhancing tumorigenicity. Current evidence suggests that this change to an exhausted state is a safety mechanism to prevent CD8+ T cells from hyperactivity and resulting cell death.^81,82^ Thus, despite chemoattractants recruiting undifferentiated CD8+ T cells to tumor sites, infiltrated CD8+ T cells face additional challenges within the TME that limit immunological efficacy.

Another barrier to CD8+ T cell TME infiltration is a slew of biophysical elements. Generally, CD8+ T cells have a modest reaction to increased stiffness and do not dramatically change their phenotype; however, a 2017 study found that within the context of stiffened arterial walls, increased stiffness was correlated with higher subsets of CMV pp65‐specific responses and CD57+ within the CD8+ population.^83^ These markers suggest increased cytotoxic activity, maturation, and decreased proliferative behaviors^84,85^ [Fig. 2(b)]. When compared to murine CD8+ T cells, human CD8+ cells have a less dramatic phenotypic response but do show considerable change in morphology at early time points, likely due to CD3 and CD28 markers. RNAseq further shows considerable difference in activated gene pathways in T cells when cultured on soft or stiff substrates.^86^ Cytotoxic CD8+ T cells (CTLs) showed some limited stiffness dependency as well.^87^ Increased degranulation of CTLs were seen in CTLs cultured on substrates of higher stiffnesses, suggesting deactivation and limited efficacy.

#### 3. CD4+T cells

In recent years, there has been increased attention toward the role that CD4+ T cells also play in tumorigenesis. While CD8+ T cells are more cytotoxic, CD4+ T cells play an important role in aiding CD8+ T cells. From a non-cancer immunological perspective, CD4+ T cells are instrumental in improving CD8+ T cell functionality and recruitment in multiple ways, including increasing antigen-presentation on dendritic cells.^88,89^ Moreover, CD4+ T cells also display delayed cytotoxic activity: tumor cells directly presenting with MHC-II antigen can recruit CD4+ T cells, which can both kill the cancer cells and enhance the anti-tumor response of CD8+ T cells by secreting IFNγ and TNFα.^90–93^ Thus, CD4+ T cells are an exciting actor to look at within tumor immunogenics, as they enhance the immune response of CD8+ T cells while also acting on tumor cells presenting with particular antigens. In addition to MHC-II antigen presentation serving as a chemotaxic signal for CD4+ T cells, evidence suggests that tumor cells may recruit CD4+ T cells through cytokines IL-10 and TGF-β: specifically, IL-17 expression in mouse colorectal tumors limited the expression of these cytokines, decreasing CD4+ T cell infiltration^94^ [Fig. 2(a)].

However, recent evidence suggests that the overlapping T cell receptors (TCRs) of CD4+ T cells and Tregs contribute to immunosuppression and poorer cancer outcomes, specifically including inhibition of CD8+ T cell activity.^89,95^ The tendency of components of the TME, including TAMs, to support conversion of CD4+ T cells into Tregs underscores the sensitivity of CD4+ T cells as an antiviral agent against tumors.^96^

In terms of mechanically sensing their environment, CD4+ T cells tend to respond more dramatically than CD8+ T cells.^97^ Antigen sensitivity for CD4+ T cells has been shown to increase as the stiffness of APC/DC increases.^87^ For primary human CD4+ T cells, activation also increased with stiffness [Fig. 2(b)]. Some activation markers seem to be more sensitive to higher stiffness, with many studies pointing to TCR/CD3 as the most critical complex involved in sensing and reacting to stiffer substrates.^97–99^ The motivation for identifying the significance of stiffness for T cell activation lies in the fact that DCs, the main APC for T cell activation, have been shown to stiffen over time.^100^

Some studies have argued that TCRs act as mechanosensors in identifying and responding to increased surface stiffness, following the engagement of cognate pMHC.^101^ The TCR-pMHC bond may experience forces and shear stress, causing conformational changes within the beta chain of the TCR.^87^ This has yet to be confirmed, but following increased IL-2 production of CD4+ naive cells when exposed to increased stiffness suggests activation and increased differentiation.

Two-dimensional and three-dimensional models of the ECM have been shown to change T cell phenotype and differentiation. A recent study cultured T cells on fast- and slow-relaxing viscoelastic hydrogels over time and measured differentially expressed genes between the conditions. Researchers found that the different T cell phenotypes were tuned via the activator-protein-1 signaling pathway.^102^

Ultimately, CD4+ T cells are a critical component of the adaptive immune response to tumor cells due to their ability to enhance the system-wide immune response, including recruitment and activity of CD8+ T cells; however, the attenuation of CD4+ T cell activity within the TME through cellular conversion to Tregs highlights the ability of the TME to avoid the immune response.

#### 4. General limitations of immune response to tumors

The ambiguous, at times unpredictable, results of chemokine presentation by tumors—particularly those of the CCL and CXCL families—make chemokine-mediated cancer interventions more difficult to predict. Moreover, the concentration of adaptive immune cells relative to innate immune cells is variable across genders, further complicating the universality of immune-based cancer interventions.^103,104^ Despite the complex biochemical signals and biophysical properties, T cell-based therapy remains a promising area of interest, especially compared to components of the innate immune response that have more varied effects on tumors. Such evidence indicates that CD8+ and CD4+ T cells are an important and effective component of the adaptive immune response to tumor cells, with recent efforts working to overcome limitations on their effectiveness.

## III. APPLICATIONS TO CANCER THERAPEUTICS: IMPROVING IMMUNE CELL INFILTRATION TO OPTIMIZE TUMOR OUTCOMES

### A. Overview of CAR-T cell therapy

In recent years, the development of genetically engineered adoptive cell therapy (ACT) has shifted the focus of immunotherapy research and shown promising results when applied to hematologic cancers. ACT refers to several technologies wherein genetic elements of lymphocytes (namely, T-cells and NK cells) can be altered to facilitate migration and show improved tumor antigen recognition.^105^ CARs (chimeric antigen receptors) are one such technology where synthetic receptors can be “programmed” into lymphocytes, thereby redirecting them and heightening their abilities to recognize and eliminate cells expressing the target antigen. The invention of these technologies has provided a novel method to address many of the physical, genetic, and biochemical defenses that malignant tumors assemble to protect themselves.

Though CAR-T cell therapy is a compelling intervention, recent works have uncovered limitations to the technology. Primarily, CAR-T cell therapy can malfunction due to antigen escape wherein tumors begin to develop resistance to single and even multiple antigen targeting CARs.^105,106^ Moreover, the technology is still associated with a high rate of toxicities—including cytokine-release syndrome, macrophage activation syndrome, immune effector cell-associated neurotoxicity syndrome, and hemophagocytic lymphohistiocytosis.^107,108^

Of particular relevance to this review, a critical issue facing CAR-T cell therapy is the threat of the solid tumor. Solid tumors do not homogeneously express antigens, making it especially difficult for CARs or methods of identifying and infiltrating tumors. Inconsistent infiltration of immune cells in solid tumors is not a limitation specific to CAR-T cells. Some tumor types tend to be “hot” (having been infiltrated by immune cells) while some are more likely to be “cold” (lacking beneficial immune cells or containing cells that block immune responses).^109^ Thus, CAR-T cell therapy faces different barriers to efficacy across solid tumor types. Moreover, CAR-T cells often fail from low T cell expansion and short-term T cell persistence within the TME, leading to T cell exhaustion: the inhibition of T cell proliferation and effectiveness due to continued antigen stimulation.^110–112^

Here, we turn our attention to recent developments that have sought to address the biophysical limitations of CAR-T cell therapy caused by the TME. Specifically, we focus on developments in vascular and perfusion mechanisms and extracellular matrix mechanisms.

### B. Coupling biophysical considerations with CAR-T cell therapy for improved infiltration

In addition to the potential of CAR-T cell therapy as a stand-alone therapeutic, recent innovations focused on manipulating the biophysical aspects of the TME have also shown substantial promise as interventions. Here, we outline recent approaches (summarized in Table I) that target biophysical factors limiting CAR-T cell efficacy, which may be coupled with the therapy to produce more robust treatments.

**TABLE I.** Therapeutic strategies to address biophysical barriers to CAR-T efficacy. Biophysical considerations could be paired with CAR-T cell therapy in order to mitigate the biophysical characteristics of solid tumors that impede the infiltration and activity of T cells. Recent efforts by researchers to overcome biophysical barriers are summarized here.

Biophysical barriers	Therapeutic strategies
Compression of blood vessels due to solid stress	Vascular normalization leads to increased vessel diameter, density, and decreases tortuosity (e.g., anti-VEGF antibodies)^116,117^
	Depleting CAFs via inhibition of the sonic hedgehog pathway (e.g., Saridegib)^120^
Elevated interstitial fluid pressure (IFP) due to vessel leakiness	Inducing an inflammatory response and vascular leakage (e.g., recombinant human TNF-α)^125^
	Lowering IFP by breaking down the breaking down the ECM via microwave ablation^127^
Hypoxia-induced immunosuppression	Modulating oxidative metabolism, thereby rescuing CD8+ T cells from hypoxia-induced apoptosis (e.g., Metformin)^130^
ECM stiffness	Suppress CAFs thereby decreasing matrix-cross-linking (e.g., Tranilast)^136^
Immunosuppressive effects of high-density ECMs	Decrease fibrosis by interfering with endothelin A (e.g., Bosentan)^136^
	Antagonize endothelin B receptors to increase adhesion of T cells to the vessel wall (e.g., Bosentan)^137^

#### 1. Addressing limitations in vasculature and perfusion

Solid stresses arise in tumors due to the physical forces associated with tumor growth. This stress pushes on surrounding tissues and results in the accumulation of stress inside the tumor.^113^ This increased stress can result in compression of lymphatic and blood vessels in the tumor, thus, reducing blood flow and the efficacy of intravenous immunotherapy.^114^ For this reason, drugs that can target the biophysical features of the tumor to reduce this stress and open the vasculature show promise in improving CAR-T cell therapy delivery to tumors.

Treatment to normalize tumor vasculature restores perfusion to improve the efficiency of the supply of therapies to the tumor. Vascular normalization involves morphological changes that include an increase in vessel diameter and density, and a decrease in tortuosity.^115^ Low doses of anti-VEGFR2 antibody have been found to accomplish normalization and decrease hypoxia by stimulating the vascular endothelial growth factor (VEGF).^116^ Thus, anti-VEGFR therapies could be promising in conjunction with immunotherapies to enhance effectiveness by increasing the availability of CAR-T cell therapy to the tumor interior. Indeed, anti-VEGF antibody (B20) used in conjunction with CAR-T cells improved survival, improved CAR-T infiltration in solid tumors, and increased the number of CD8+ T cells in the TME.^117^

#### 2. Addressing limitations in CAFs

Alternatively, immunotherapy efficacy could be improved by depleting CAFs (through inhibition of the sonic hedgehog pathway), thereby increasing perfusion through reduced solid stress and compression of blood vessels.^118^ Inhibition of the sonic hedgehog pathway can be accomplished with Saridegib, a drug that blocks a receptor in the pathway.^119^ Already, Saridegib has been used to great effect: treatment led to a 10% increase in the diameter of blood and lymphatic vessels and a 47% increase in the fraction of perfused blood vessels in transplanted pancreatic tumors.^120^ For this reason, Saridegib may be effective in conjunction with CAR-T cell therapy for improved delivery. In a phase 1 clinical trial of Saridegib, the drug was used alone on solid tumors resistant to standard therapy. Of the 55 evaluable patients with non-basal cell carcinoma solid tumors, 53% achieved stable disease. Basal cell carcinoma responded with 8 of 28 patients achieving an objective response (2 complete response, 6 partial response).^121^

#### 3. Addressing limitations in interstitial fluid pressure

Having explored how tumor stiffness impedes drug perfusion by constricting vessels, it is important to note that interstitial fluid pressure (IFP) also plays a role. Tumor vessels are typically leakier than healthy vessels, resulting in an elevated interstitial fluid pressure.^122^ Elevated IFP may then impede the effectiveness of immunotherapies by reducing the penetration of intravenous drugs into solid tumors.^123^ Thus, reducing IFP through vascular disrupting agents may improve drug delivery.

TNF-α is involved in systemic inflammatory responses and causes vascular leakage. However, it is severely toxic, and therefore, has not been widely utilized in clinical settings.^124^ To address this, Libutti *et al.* synthesized PEGylated colloidal gold nanoparticles to carry recombinant human TNF-α and found that it successfully targeted tumor cells, reduced IFP, and remained nontoxic until doses three times higher than systemically administered TNF-α.^125^ Furthermore, the particles, when combined with chemotherapy, resulted in improved survival and lower tumor burden.^125^ Thus, drugs that lower IFP appear promising in improving treatment delivery to solid tumors, perhaps including CAR-T cell therapy. Unfortunately, higher endogenous TNF-α levels after CAR-T therapy have been associated with severe bleeding.^126^ Therefore, safer avenues to normalize interstitial pressure could be promising.

A study of microwave ablation (MWA) used in conjunction with CAR-T cells found that the microwave ablation reduced IFP, improving tumor permeability and T cell infiltration.^127^ MWA works to break down the TME, as shown by decreased hyaluronic acid levels, a main component of the ECM, after treatment. In addition, MWA increased the partial pressure of oxygen, thereby alleviating immunosuppressive hypoxia. In combination with CAR-T therapy, MWA led to better tumor regression than either treatment alone. This is perhaps because MWA assists CAR T cell infiltration and reduces their exhaustion as shown by elevated IL-2, IFN-gamma, and TNF-α levels.^127^

#### 4. Addressing limitations in hypoxia

In addition, immunotherapy efficacy in solid tumors is impeded by hypoxia-induced immunosuppression as a result of reduced perfusion. The hypoxia associated with vessel compression can impede effector immune cells in killing tumor cells as well as reprogramming tumor-associated macrophages from immuno-supportive to immunosuppressive phenotypes.^128^

Hypoxia appears to play a role in resistance to immunotherapy, particularly anti-PD-1 therapy. In particular, oxidative metabolism in tumors results in intratumoral hypoxia, thereby decreasing CD8+ T cell infiltration and reducing the efficacy of the immunotherapy drug.^129^ Thus, drugs that modulate oxidative metabolism (i.e., Rosiglitazone) may be promising in increasing immunotherapy efficacy in the context of CAR-T cell therapy by improving CD8+ T cell viability.

One possible treatment to improve CD8+ T cell viability and, therefore, CAR-T cell therapy efficacy is Metformin. The drug is a type 2 diabetes treatment that has been found to diminish intratumoral accumulation of myeloid-derived suppressor cells and downregulate PD-L1 expression in tumor cells.^130^ When mice with hypoxic tumors were treated with metformin and tumor-specific CD8+ T cells, Metformin was found to rescue CD8+ T cells from hypoxia-induced apoptosis and improve infiltration into hypoxic areas of the tumor without actually reducing tumor hypoxia.^130^

#### 5. Addressing limitations in extracellular matrix stiffness and density

Increased ECM stiffness and density in tumors are in part a result of the activation of fibroblasts to form CAFs, which further increase the production of matrix-cross-linking enzymes in the ECM and contribute to fibrosis.^131,132^ By compressing tumor vessels and preventing the perfusion of drugs through the tumor, this increased production drives solid stress that impedes the efficacy of immunotherapy.^133^

Mechanotherapeutics, despite not being directly toxic to the tumor, can reduce tumor stiffness and improve immunotherapy function. Tranilast, an antihistamine drug, was found to soften tumors in a dose-dependent manner by suppressing CAFs, improving the normalized perfused area by three times.^128^ Thus, Tranilast could be effective when used in conjunction with CAR-T cell therapy by increasing the infiltration of CAR-T cells into solid tumors. Indeed, Tranilast has been found to inhibit tumor growth and fibrosis as well as promote CD8+ T cell infiltration.^134^

High collagen concentration is also associated with reduced T cell proliferation and more CD4+ cells relative to infiltrative CD8+ T cells.^135^ In addition, whole-transcriptome analysis reveals that high ECM density also downregulates cytotoxic activity and upregulates regulatory T cell markers.^135^ Thus, high-density ECMs appear to have immunosuppressive effects, thereby reducing the efficacy of CAR-T cell therapy.

Mechanotherapeutics that reduce ECM density may, therefore, be an effective route to reduce these immunosuppressive effects. One promising drug is Bosentan, which was found to reduce stiffness and hypoxia by interfering with the endothelin A receptor that induces fibrosis.^136^ Alone, Bosentan reduces tumor stiffness but does not affect tumor growth rate. However, in a cocktail of anti-PD-1 and anti-CTLA-4 antibodies, it improves immune checkpoint blockade therapy efficacy, as shown by reduced tumor volume and increases survival rates in mice.^136^ Interestingly, Bosentan is a nonselective endothelin receptor blocker, meaning it also antagonizes endothelin B receptors. This antagonism increases T cell adhesion to the vessel wall by upregulating adhesion molecules on circulating T cells.^137^ Thus, Bosentan may improve CAR-T cell therapy by improving the perfusion of T cells from the blood to the tumor as well as improving perfusion through the tumor by inhibiting fibrosis. Indeed, treatment with Bosentan increased colocation of T cells and endothelial cells and improved T cell penetration to the center of solid tumors.^136^ In addition, when used in conjunction with immune checkpoint blockade therapy, Bosentan increased T cell activity as evidenced by increased proliferation of CD8+ T cells.^136^ It remains to be seen whether combined CAR-T cell and Bosentan therapy improves CAR-T cell efficacy, though current knowledge suggests that the reduced stiffness created by Bosentan could improve CAR-T cell infiltration.

## IV. CONCLUDING REMARKS

Many characteristics of the TME impact the ability for tumor cells to proliferate, metastasize, resist treatment, and thrive, including matrix stiffness and porosity. The ECM remodeling typical of tumors is mediated by cell communication and hypoxia and is relevant to immune function since it impedes T cell infiltration. Unfortunately, this, in addition to biochemical factors that impair immune cell activation, weakens the anti-tumor response of both innate and adaptive immune responses. In particular, cytotoxic CD8+ T cells become exhausted and have difficulty penetrating solid tumors. Thus, biophysical and biochemical aspects of T cell interactions with the TME greatly influence the efficacy of treatments like CAR-T cell therapy. Barriers, such as solid stress, reduced perfusion, hypoxia, and ECM density, inhibit CAR-T cell therapy. Mechanotherapeutics that address these barriers show promise when used in conjunction with CAR-T cell therapy.

## Data Availability

Data sharing is not applicable to this article as no new data were created or analyzed in this study.
